# Deletion of the L-Lactate Dehydrogenase Gene *ldh* in *Streptococcus pyogenes* Leads to a Loss of SpeB Activity and a Hypovirulent Phenotype

**DOI:** 10.3389/fmicb.2017.01841

**Published:** 2017-09-21

**Authors:** Sonja Oehmcke-Hecht, Leif E. Nass, Jan B. Wichura, Stefan Mikkat, Bernd Kreikemeyer, Tomas Fiedler

**Affiliations:** ^1^Institute of Medical Microbiology, Virology, and Hygiene, Rostock University Medical Centre Rostock, Germany; ^2^Core Facility Proteome Analysis, Rostock University Medical Centre Rostock, Germany

**Keywords:** SpeB, LDH, *Galleria mellonella*, *Streptococcus pyogenes*, contact system

## Abstract

*Streptococcus pyogenes* uses lactic acid fermentation for the generation of ATP. Here, we analyzed the impact of a deletion of the L-lactate dehydrogenase gene *ldh* on the virulence of *S. pyogenes* M49. While the *ldh* deletion does not cause a general growth deficiency in laboratory media, the growth in human blood and plasma is significantly hampered. The *ldh* deletion strain is furthermore less virulent in a *Galleria mellonella* infection model. We show that the *ldh* deletion leads to a decrease in the activity of the cysteine protease SpeB, an important secreted virulence factor of *S. pyogenes*. The reduced SpeB activity is caused by a hampered autocatalytic activation of the SpeB zymogen into the mature SpeB. The missing SpeB activity furthermore leads to increased plasmin activation and a reduced activation of the contact system on the surface of *S. pyogenes*. All these effects can be reversed when *ldh* is reintroduced into the mutant via a plasmid. The results demonstrate a previously unappreciated role for LDH in modulation of SpeB maturation.

## Introduction

*Streptococcus pyogenes* (group A streptococcus, GAS) is an important human pathogen. It is equipped with a large number of virulence factors. The expression of these virulence factors is tightly controlled by a complex network of regulatory proteins and sRNAs (Fiedler et al., 2010; Patenge et al., 2013; Walker et al., 2014). Taxonomically, GAS belongs to the Lactobacillales meaning that it converts carbohydrates to lactic acid. In lactic acid bacteria, the major enzyme responsible for pyruvate degradation and recycling of the NAD^+^ reduced during glycolysis is L-lactate dehydrogenase (Fiedler et al., 2011; Levering et al., 2012, 2016; Feldman-Salit et al., 2013). In a previous study, we could show that deletion of the L-lactate dehydrogenase gene in GAS M49 strain 591 does not affect the growth of the bacteria in complex or chemically defined laboratory media. The bacteria are simply switching their metabolism from homofermentative lactate production to the mixed acid branch with production of ethanol, acetate, formate, and an additional ATP per glucose (Fiedler et al., 2011). Here, we show that the deletion of the *ldh* gene in GAS M49 strain 591 leads to a significant loss of fitness in human blood or plasma, a decreased contact system activation on the bacterial surface, an increased plasmin/streptokinase (Ska) activity and decreased virulence in a *Galleria mellonella* infection model. We show that this phenotype can be explained by the loss of activity of the streptococcal cysteine protease SpeB in the *ldh* deletion strain.

The GAS protein originally named streptococcal pyrogenic exotoxin B (SpeB) is neither pyrogenic nor is it an exotoxin. Instead, it is a potent secreted cysteine protease and an important virulence factor in GAS (Nelson et al., 2011). The *speB* gene is transcribed as a bicistronic mRNA with the *spi* gene encoding the SpeB inhibitor protein Spi (Kagawa et al., 2005). Intracellularly, Spi probably prevents SpeB from cleaving cytoplasmic GAS proteins (Kagawa et al., 2005). Extracellularly, SpeB is activated from its 40 kDa zymogen into an active 28 kDa enzyme by autocatalytic cleavage under reducing conditions (Doran et al., 1999). The mechanisms triggering the activation process are not fully understood. There is experimental evidence that cell wall-anchored M protein is involved in the activation of SpeB into the mature enzyme (Collin and Olsén, 2000). SpeB has been shown to cleave immunoglobulins, complement factors, and numerous host matrix and plasma proteins (Kapur et al., 1993a,b; Herwald et al., 1996; Collin and Olsén, 2001; Terao et al., 2008; Honda-Ogawa et al., 2013). Among the plasma proteins degraded by SpeB is high molecular weight kininogen (HK), a component of the human contact system (Herwald et al., 1996). The contact system, also known as the intrinsic pathway of coagulation, consists of four proteins, factor XI, factor XII (FXII), plasma kallikrein (PK) and HK (Frick et al., 2006). FXII is activated on negatively charged surfaces. Activated FXII activates (i) factor XIa which triggers the intrinsic pathway of coagulation, and (ii) prekallikrein into PK which cleaves HK into kinins, e.g., the proinflammatory bradykinin, and smaller peptides, e.g., the antimicrobially active NAT26 (Frick et al., 2006). Prekallikrein can also be activated by the plasma protease plasmin. The activation of plasminogen into plasmin is usually mediated by human tissue or urokinase plasmin activators tPA and uPA, but can also be activated via Ska, another secreted GAS virulence factor (Nitzsche et al., 2015). SpeB potently degrades Ska, thereby reducing plasmin activity (Svensson et al., 2002). Furthermore, bacteria can directly interact with different contact system components (Nickel and Renne, 2012). GAS can bind HK via the M protein and cleave it via SpeB (Ben Nasr et al., 1997). Hence, there is a complex network of interactions of GAS with coagulation factors and, consequently, with hemostasis in the human host.

## Materials and Methods

### Bacterial Strains and Culture Conditions

The *S. pyogenes* serotype M49 wild type (WT) strain 591 was obtained from R. Lütticken (Aachen). The L-lactate production deficient mutant (Δ*ldh*) of *S. pyogenes* M49 has been described previously (Fiedler et al., 2011). Generally, bacteria were grown in Todd Hewitt broth (Oxoid) supplemented with 0.5% yeast extract (THY; Oxoid) at 37°C in a 5% CO_2_/20% O_2_ atmosphere.

### Genetic Manipulations

For the construction of a complementation plasmid a fragment comprising the *ldh* gene and its native promoter (280 bp upstream of the start codon) of the *S. pyogenes* M49 591 WT strain was amplified by PCR using a Phusion High Fidelity DNA polymerase. This fragment was inserted into the shuttle vector pAT19 (Trieu-Cuot et al., 1991) via *Bam*HI and *Sal*I restriction sites. Correct insertion of the fragment was confirmed by sequencing. The resulting plasmid was transferred into the *S. pyogenes* M49 591 *ldh* deletion strain via electroporation. The resulting strain *S. pyogenes* M49 591Δldh::ldh was tested for LDH activity to confirm functional expression of the plasmid-located *ldh* gene.

### Measurement of LDH Activity

For measurements of LDH activity, *S. pyogenes* strains were grown in THY under standard conditions. At mid-exponential growth phase (OD at 600 nm = 0.5) cells of 2 ml culture were pelleted by centrifugation and washed twice in sodium phosphate buffer (50 mM, pH 6.8). Intracellular proteins were released by enzymatic degradation of the cell wall with phage lysin PlyC for 10 min at 37°C as described previously (Nelson et al., 2006; Koller et al., 2008). Subsequently, lysates were centrifuged for 10 min at 20,000 *g* and 50 μl of the supernatants were applied in the activity assay either directly or in a 1:25 dilution in sodium phosphate buffer. LDH activity was determined via measurement of the conversion of NADH to NAD^+^ as described previously (Levering et al., 2012). In brief, the reaction mixture contained 0.5 mM fructose-1,6-bisphosphate, 0.17 mM NADH, 10 mM sodium pyruvate and 50 μl sample in 50 mM sodium phosphate buffer (pH 6.8) in a total volume of 1 ml and the absorption at 340 nm was measured for 5 min. The specific activity is given in units per milligram protein, where 1 unit is defined as conversion of 1 μM NADH per minute. Protein concentrations in the *S. pyogenes* lysates were measured with the Pierce^TM^ Coomassie (Bradford) Protein Assay Kit (Thermo Fisher Scientific).

### Proteome Analysis by NanoLC-HDMS^E^

In-solution digestion of proteins with trypsin in sodium deoxycholate-containing buffer was performed as previously described (Masuda et al., 2008; Pade et al., 2017) using total protein extracts prepared in a Precellys 24 homogenizer (peqLab Biotechnologie GmbH, Erlangen, Germany). Mass spectrometry was performed on a Synapt G2-S mass spectrometer (Waters, Manchester, United Kingdom) coupled to a nanoAcquity UPLC system (Waters) as described (Pade et al., 2017). In short, peptides mixtures were separated on an analytical column (ACQUITY UPLC HSS T3, 1.8 μm, 75 μm × 250 mm, Waters) at a flow rate of 300 nl/min using a gradient from 3 to 32% acetonitrile in 0.1% formic acid over 150 min. The SYNAPT G2-S instrument was operated in data-independent mode (Geromanos et al., 2009), characterized by parallel fragmentation of multiple precursor ions in combination with ion-mobility separation as an additional dimension of separation (referred to as HDMS^E^) (Shliaha et al., 2013; Distler et al., 2014). Samples were measured in duplicate.

### NanoLC-HDMS^E^ Data Processing, Protein Identification, and Quantification

Progenesis QI for Proteomics version 2.0 (Nonlinear Dynamics, Newcastle upon Tyne, United Kingdom) was used for raw data processing, protein identification, and label free quantification. For the database search a database containing 1701 protein sequences from *S. pyogenes* serotype M49 (strain NZ131) (UniProt release 2016_05) appended with the sequences of rabbit phosphorylase B (P00489) and porcine trypsin was compiled. One missing cleavage site was allowed, oxidation of methionine residues was considered as variable modification, and carbamidomethylation of cysteines as fixed modification. The false discovery rate based on the search of a reversed database was set to 1%. Peptides were required to be identified by at least three fragment ions and proteins by at least seven fragment ions and two peptides. With the subsequent filtering steps peptides were removed that had (i) a peptide score below 5.96, (ii) a mass error above 10 ppm, (iii) less than six amino acid residues. Proteins were quantified by the absolute quantification Hi3 method using Hi3 rabbit phosphorylase B Standard (Waters) as reference (Silva et al., 2006). Protein abundance changes by a factor of at least two, accompanied by ANOVA *p*-values <0.05 for the comparison between the respective groups were regarded as significant.

### Survival Assays

The survival assays in human blood or plasma were performed as described previously (Nakata et al., 2009). In short, bacteria grown in THY medium were harvested in the exponential growth phase (optical density at 600 nm of 0.4–0.5). Bacteria were suspended in phosphate-buffered saline (PBS), the optical density at 600 nm was adjusted to 0.3 and this suspension was diluted 1:10,000 in PBS. The viable counts were determined by plating serial dilutions on THY agar plates. Twenty microliters of the bacterial suspension were inoculated into 480 μl of fresh citrated human blood or plasma and incubated for 3 h at 37°C with rotation. The viable counts were determined by plating serial dilutions on THY agar plates and related to the inoculum.

### Ethics Approval Statement

The protocol for the collection of human blood for the blood and plasma survival assays was approved by the Ethikkommission an der Medizinischen Fakultät der Universität Rostock (ethics committee vote: A 2014-0131). The experiments were conducted in accordance with the ICH-GCP guidelines. Informed consent was obtained from all subjects.

### Western Blot Analysis

For sampling, overnight cultures were set to 2 × 10^8^ CFU/ml and 250 μl were mixed with equal amount of human normal plasma and incubated at 37°C for 60 min with shaking (600 rpm). Incubation of plasma with PBS, bacteria with PBS and plasma with kaolin served as controls. Cells were pelleted by centrifugation, washed three times in PBS, suspended in 100 μl glycine (0.1 M) and incubated at room temperature for another 10 min. Cells were pelleted by centrifugation and supernatants were neutralized by the addition of 20 μl Tris–HCl (1 M, pH 8.4). A total of 100 μl of the suspensions were mixed with 20 μl SDS sample buffer (5×). SDS-PAGE was performed as described earlier (Neville, 1971). Following SDS-PAGE, separated proteins were transferred onto nitrocellulose membranes. Western blot analyses were performed with sheep antibodies against HK (1:3,000; Affinity Biologicals) and its degradation products as described previously (Mattsson et al., 2001). Chemiluminescence was detected on a Kodak ID3.5 Image Station 440CF.

### Dot Blot Analysis

For dot blot analysis, 100 μl of filter-sterilized (0.2 μm) supernatants (either pure or diluted with fresh THY) of overnight cultures of the bacteria in THY medium were transferred on a nitrocellulose membrane using a Bio-Dot^®^ microfiltration apparatus (Bio-Rad). The detection was performed with a SpeB (bD-12) antibody from goat (1:1,000) and a donkey-anti-goat IgG-HRP secondary antibody (1:1,000; both Santa Cruz Biotechnology) as described elsewhere (Mattsson et al., 2001).

### Measurement of FXII/PK Activity

FXII/PK activity on bacterial surfaces exposed to plasma was measured using chromogenic substrate S-2302 (H-D-Pro-Phe-Arg-pNA⋅2HCl; Chromogenix). Ten-milliliter overnight cultures (THB) of the *S. pyogenes* M49 strains were washed three times with 50 mM Tris–HCl (pH 7.5) and diluted (final concentration, 3 × 10^7^ CFU/ml) in 50 mM Tris buffer. Then, 100 μl of bacterial suspension was mixed with 100 μl of plasma or buffer (control), followed by incubation at 37°C for 30 min. After centrifugation, the pellets were washed three times and suspended in 200 μl of buffer containing 1 mM substrate S-2302. After 60 min at 37°C, the samples were centrifuged, and the absorbance of the supernatants was measured at 405 nm in a microplate reader. Control values (bacteria incubated in buffer) were used as a blank. No endogenous proteolytic activity was measured when S-2303 was incubated with bacteria in the absence of plasma.

### Measurement of Plasmin Activity

To measure the plasmin activity on bacterial surfaces exposed to plasma, bacteria were grown overnight in 10 ml of THB, washed three times with PBS, and diluted (final concentration 5 × 10^7^ CFU/ml) in PBS. Next, 200-μl bacterial suspensions were mixed with 200 μl of plasma or buffer, followed by incubation for 3 h. After three further washing steps with PBS, the pellet was suspended in Tris–NaCl buffer (19.2 mM/1.062 M; pH 7.5) containing 20 μg/ml of the chromogenic substrate S-2251 (H-D-Val-Leu-Lys-pNA⋅2HCl; Sigma), followed by an incubation for 60 min at 37°C. Samples were centrifuged, and the absorbance of the supernatants was measured at 405 nm in a microplate reader.

### Measurement of SpeB Activity

For determination of SpeB activity in bacterial culture supernatants bacteria were grown for 8 h in THY (Becton, Dickinson and Company), diluted 1:100 in fresh THY medium and further cultivated for 12 h. Cells were pelleted, 1 ml per supernatant was filter-sterilized (0.2 μm pore size), and 50 μl of sterile supernatants were activated with 2 mM dithiothreitol (DTT) for 30 min at 37°C. Then, 150 μl of substrate solution (1 mM N-benzoyl-Pro-Phe-Arg-p-NA, 60 mM sodium phosphate pH 6) were added and incubated for 2 h at 37°C. Absorbance was measured immediately after substrate addition and after the 2 h incubation at 405 nm in a microplate reader. As a specificity-control, samples without DTT activation and samples with addition of 0.05 mM cysteine protease inhibitor E64 (Sigma) were measured simultaneously.

### *Galleria mellonella* Infection Model

Larvae of the greater wax moth *G. mellonella* were obtained from Reptilienkosmos (Niederkrüchten, Germany). For infection experiments, *S. pyogenes* strains were grown overnight in THY, washed twice in a 0.75% NaCl solution and suspended in 0.75% NaCl to a final concentration of 1.5–2 × 10^8^ CFU/ml. Larvae with a weight of 150–200 mg were inoculated with 10 μl of this bacterial suspension, resulting in an infection dose of 1.5–2 × 10^6^ CFU/larva. Bacteria were injected into the hemocoel of the larvae between the last pair of legs using a microapplicator (World Precisions Instruments, Sarasota, United States) and a fine dosage syringe (Omnican^®^-F, 0.01–1 ml, 0.30 × 12 mm, B. Braun AG, Melsungen, Germany). As a control, larvae were mock inoculated with 10 μl of a 0.75% NaCl solution. Survival of the larvae was observed for seven days. Larvae were regarded dead when they were not moving upon repeated physical stimulation with tweezers (Mukherjee et al., 2010).

## Results

**FIGURE 1:**
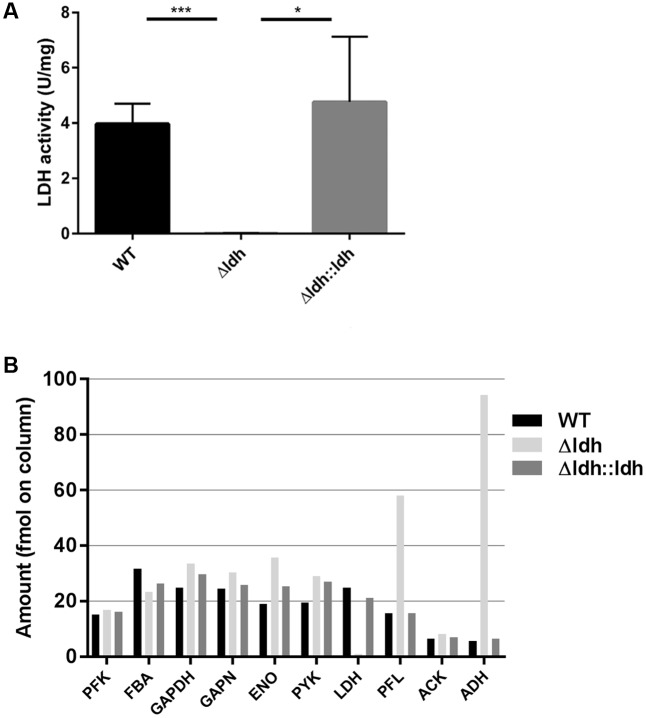
LDH-activity **(A)** and abundance of glycolytic and pyruvate branching enzymes **(B)**. LDH activity was measured in protein extracts of cultures grown to the exponential growth phase (OD_600_ = 0.5) in THY (*n* = 3, ^∗^*p* < 0.05, ^∗∗∗^*p* < 0.001, unpaired two-tailed *t*-test). For the measurement of protein abundances **(B)**, protein extracts of three biological replicates of THY cultures (OD_600_ = 0.5) were pooled and subjected to label-free protein quantification using nanoLC-HDMS^E^.

As previously reported, a deletion of the *ldh* gene in *S. pyogenes* M49 strain 591 leads to a metabolic switch from homofermentative lactate production to mixed acid fermentation but not to general growth retardation of the bacteria in laboratory media (Fiedler et al., 2011). Here we aimed to elucidate whether the loss of L-lactate dehydrogenase has an impact on the fitness of *S. pyogenes* in infection-related conditions. For that purpose we first constructed a complementation strain carrying a pAT19-based plasmid containing the *S. pyogenes* M49 strain 591 *ldh* gene with its native promoter. Protein extracts obtained from cells in the exponential growth phase (OD = 0.5) in THY medium showed the same specific LDH activity for complementation strain and WT, while extracts of the *ldh* deletion strain were completely devoid of L-LDH activity (**Figure 1A**). Furthermore, the abundance of proteins was analyzed in total extracts from exponentially growing bacteria of all three strains by label-free protein quantification using ion mobility-enhanced data-independent acquisition (HDMS^E^) (Distler et al., 2016). Protein extracts from three biological replicates per strain were pooled for these measurements. In the WT and complementation strain, pyruvate branching enzymes were at similar levels after overnight growth in THY, with relatively high abundance of lactate dehydrogenase and relatively low levels of pyruvate formate lyase and alcohol dehydrogenase as compared to the *ldh* deletion strain (**Figure 1B**). Abundance of glycolytic enzymes, i.e., phosphofructokinase, fructose-bisphosphate aldolase, glyceraldehyde-3-phosphate dehydrogenase, enolase, and pyruvate kinase, was similar in all three strains (**Figure 1B**). Hence, in terms of pyruvate branching, the complementation strain is able to restore the WT phenotype. The abundance of the enzymes encoded upstream and downstream of LDH (NADH oxidase and gyrase A) was unaffected in the *ldh* deletion strain (Supplementary Table 1).

**FIGURE 2:**
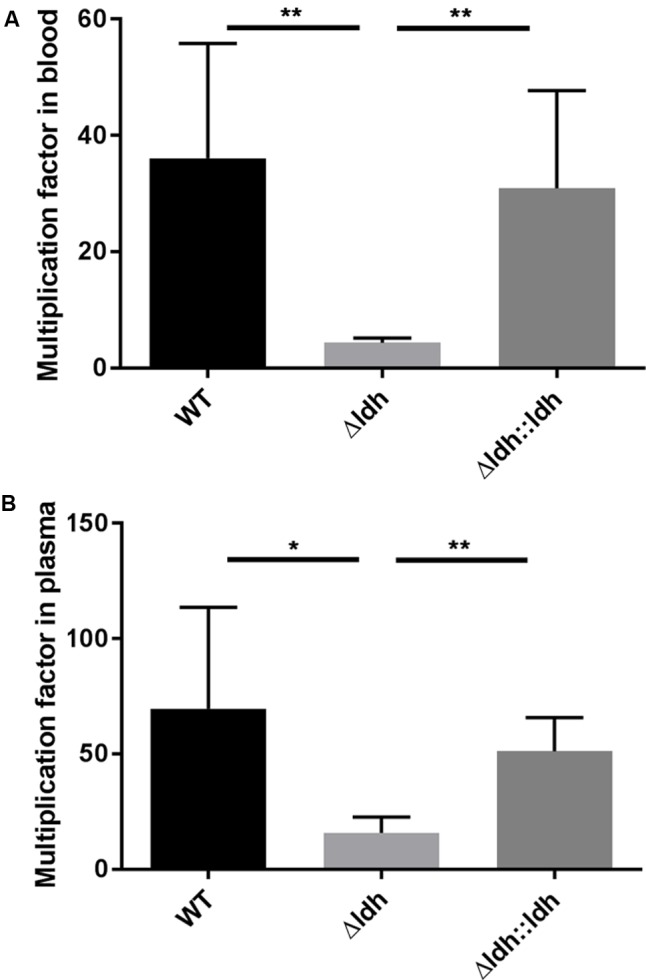
Growth in human blood **(A)** and plasma **(B)**. Bacteria were incubated in citrated blood or plasma for 3 h. Viable counts before and after the incubation were used to calculate multiplication rates (*n* ≥ 5, ^∗^*p* < 0.05, ^∗∗^*p* < 0.01, unpaired two-tailed *t*-test).

Next, we assessed the ability of WT, *ldh* deletion mutant and complementation strains to multiply in citrated human blood and plasma, respectively. As shown in **Figure 2A**, the *ldh* deletion strain had significantly lower multiplication rates in blood than WT and complementation strain. The phenotype in plasma resembled that in blood (**Figure 2B**). This indicates that not only cellular components but also soluble components such as plasma proteins seem to be responsible for the reduced multiplication of the *ldh* deletion strain.

**FIGURE 3:**
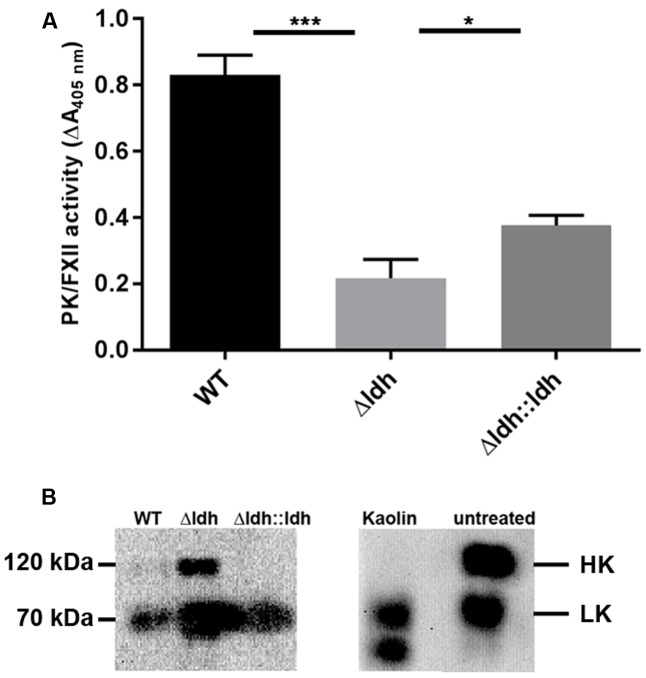
PK/FXII activity **(A)** and Western blot analysis of HK bound at the bacterial surface **(B)**. PK/FXII activity was determined in plasma samples exposed to the bacteria and depicted as rates of conversion of a chromogenic plasma kallikrein substrate (*n* = 3, ^∗^*p* < 0.05, ^∗∗∗^*p* < 0.001, unpaired two-tailed *t*-test). For Western blots, plasma proteins eluted for bacteria or kaolin (as a positive control) were applied to SDS-PAGE, blotted on nitrocellulose membranes, and detected with a kininogen-specific primary antibody and a HRP-conjugated secondary antibody.

Since the contact system is one of the humoral innate immune mechanisms that is activated by GAS M49 (Nitzsche et al., 2015), we analyzed the extent of contact activation at the surface of the bacteria. For that purpose the activity of the proteases FXII and PK bound to the bacterial surface after incubation in plasma was determined in a colorimetric assay. As depicted in **Figure 3A**, the FXII/PK activity at the surface of the *ldh* deletion strain was significantly reduced compared to the WT. As contact system activation is accompanied with a degradation of HK, we analyzed whether intact HK is bound at the surface of M49, M49_Δldh_, and M49_Δldh::ldh_ strains. For that purpose, bacteria were incubated in plasma, and, after washing, the adsorbed proteins were eluted from the surface and analyzed by western blot with antibodies directed against HK and low-molecular weight kininogen (LK). LK is a shorter splice variant of HK (Furuto-Kato et al., 1985), but has no function in contact activation (Lalmanach et al., 2010), and the polyclonal antiserum against HK also reacts with LK. Plasma alone or plasma treated with kaolin (a contact activator) served as negative and positive control, respectively. As depicted in **Figure 3B**, in untreated plasma HK could be detected at 120 kDa and LK at 66 kDa. In the kaolin-treated plasma, HK was processed and consequently the signal at 120 kDa disappeared. A similar pattern was obtained in the eluate samples from the bacteria, which contain plasma proteins adsorbed at the surface of *S. pyogenes* and its mutants (**Figure 3B**). The 120 kDa HK signal was completely absent in eluate samples from the WT and the complemented strain (**Figure 3B**). This is in contrast to the *ldh* deletion strain, where intact 120 kDa HK was eluted from the bacterial surface (**Figure 3B**). This implies that the *ldh* deletion strain does not degrade surface-bound HK, which is in line with its lower surface FXII/PK activity as described above (**Figure 3A**).

**FIGURE 4:**
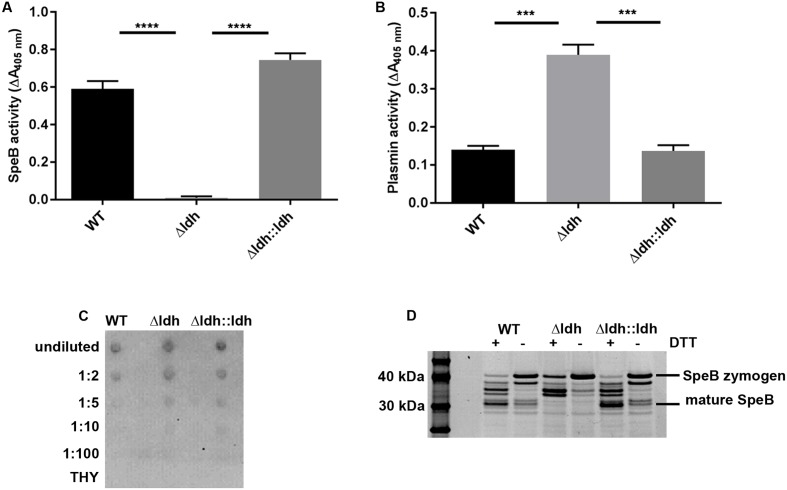
The *ldh* deletion has an impact on SpeB and plasmin activity. SpeB activity **(A)** was determined in culture supernatants of overnight cultures in THY (*n* = 4, ^∗∗∗∗^*p* < 0.0001, unpaired two-tailed *t*-test), plasmin activity **(B)** was measured on the surface of bacteria after incubation in pooled human plasma (*n* = 3, ^∗∗∗^*p* < 0.001, unpaired two-tailed *t*-test), dot blot analysis of SpeB abundance in culture supernatants with SpeB specific antibodies **(C)**, non-reducing SDS-PAGE showing SpeB activation via DTT treatment **(D)**.

A major streptococcal protein interacting with the contact system is the secreted cysteine protease SpeB (Nelson et al., 2011) as it can degrade HK into kinins (Herwald et al., 1996). On the other hand SpeB also degrades bacterial virulence factors such as Ska and M protein (Walker et al., 2014). Therefore, we determined SpeB activity in the supernatants of THY cultures (**Figure 4A**). In the supernatant of the *ldh* deletion strain no SpeB activity was detectable. In contrast, in the supernatants of WT and complementation strain significant SpeB activity was measured (**Figure 4A**). Both—PK and SpeB—efficiently cleave HK (Herwald et al., 1996), thus the completely restored SpeB activity in the complemented strain probably induces the complete HK degradation, although FXII/PK levels are not fully restored in the complemented strain (**Figure 3B**). In line with the absence of SpeB activity, the *ldh* deletion strain showed a significantly higher surface plasmin activity after incubation in human plasma compared to WT and complementation strain (**Figure 4B**). The loss of SpeB activity in the *ldh* deletion strain is apparently not due to reduced SpeB production, as in dot blot analysis with SpeB-specific antibodies SpeB could be detected in the supernatants of all three strains (**Figure 4C**). The reason for the absence of SpeB activity in the culture supernatants of the *ldh* deletion strain rather seems to be a disturbed autocatalytic activation of the 40 kDa SpeB zymogen into the 28 kDa mature SpeB. In the culture supernatant of the *ldh* deletion mutant there was only a faint signal for the mature 28 kDa SpeB detectable. Instead, intermediates frequently occurring in SpeB maturation accumulated (Doran et al., 1999; Chen et al., 2003) (**Figure 4D**). In contrast, in supernatants of WT and complementation strain the mature SpeB was much more abundant than in the *ldh* deletion strain (**Figure 4D**).

**FIGURE 5:**
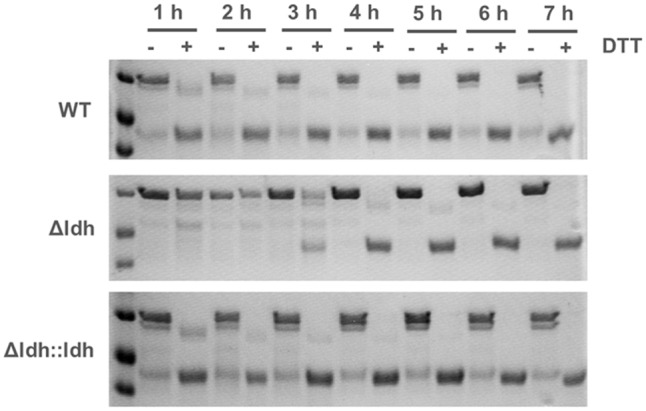
Impact of the *ldh* deletion on SpeB maturation. SpeB maturation in the culture supernatants of wild type, *ldh* deletion and complementation strain in the presence of DTT over 7 h was analyzed via SDS-PAGE.

Sequencing of the *speB* locus in all three strains revealed no mutations in the promoter region or the coding sequence of the *speB* gene. In time-course experiments with exposure of the culture supernatants of all three strains to DTT for up to 7 h, we observed that in WT and complementation strain, the SpeB zymogen is almost completely converted into the mature SpeB after 1 h (**Figure 5** and Supplementary Figure 1). In contrast, a visible accumulation of mature SpeB in the culture supernatants of the *ldh* deletion strain could only be observed after 3–4 h incubation under reducing conditions (**Figure 5** and Supplementary Figure 1). The addition of 30 mM L-lactate to the culture supernatant of the mutant prior to the DTT activation did not lead to faster maturation of SpeB, indicating that missing lactate in the supernatants is not responsible for the delay in SpeB activation (see Supplementary Figure 2).

The streptococcal endopeptidase HtrA (aka DegP) has previously been reported to support the maturation of SpeB (Lyon and Caparon, 2004; Rosch and Caparon, 2005). Furthermore, cell-wall anchored M protein has been described to be necessary for maturation of SpeB in a M1 serotype GAS strain (Collin and Olsén, 2000). In our proteomic data, we, however, found HtrA abundances unchanged in the *ldh* deletion strain compared to WT and complementation strain (see Supplementary Table 1). The M protein amount in WT and *ldh* deletion strain was similar as well, while in the complementation strain a moderately increased M protein amount was measured (see Supplementary Table 1).

**FIGURE 6:**
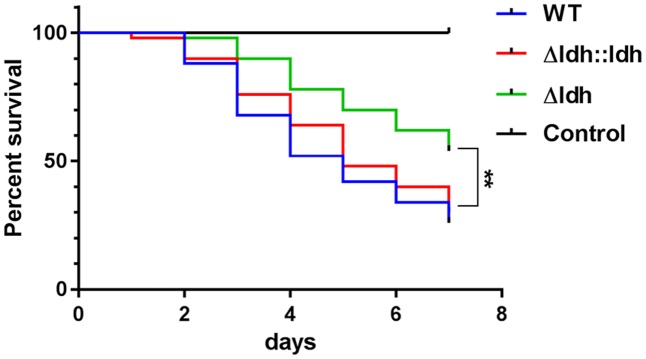
*Galleria mellonella* infection model. Survival of *G. mellonella* larvae after infection with WT, *ldh* deletion and complementation strains (*n* = 50, ^∗∗^*p* < 0.01, log-rank test).

To elucidate the impact of the *ldh* deletion on virulence of the M49 strain 591 *in vivo*, larvae of the greater wax moth *G. mellonella* were used as model organisms. *G. mellonella* is an easy-to-handle and well-established invertebrate infection model organism that reliably reflects differences in virulence of *S. pyogenes* as they can also be observed in more complex mammalian model organisms (Olsen et al., 2011; Cook and McArthur, 2013; Loh et al., 2013). Here, the larvae were inoculated with 1.5–2 × 10^6^ CFU/animal by injecting the bacterial suspension into the hemocoel of the larvae. As controls, larvae were mock-inoculated with an equal volume of a sterile physiological NaCl solution. Survival of the larvae was followed for 7 days after infection. Larvae infected with the *ldh* deletion mutant survived the 7-day period in a significantly higher proportion (54% living animals at day 7) than larvae infected with the WT (26%) or complementation strain (30%) (**Figure 6**). Hence, the reduced virulence of the *ldh* deletion strain *in vitro* is also displayed *in vivo* in the *Galleria* infection model.

## Discussion

The secreted streptococcal cysteine protease SpeB is one of the central virulence factors of GAS. SpeB is associated with an invasive phenotype of the bacteria (Carroll and Musser, 2011). SpeB degrades host proteins as well as proteins secreted by GAS (Nelson et al., 2011). After biosynthesis, cytosolic SpeB is probably kept inactive by the SpeB inhibitor protein Spi, which is encoded on a bicistronic operon with the *speB* gene (Kagawa et al., 2005). During secretion of SpeB via the ExPortal protein secretion microdomain located Sec translocon of *S. pyogenes* (Rosch and Caparon, 2004, 2005), the leader peptide is cleaved off, and the protein is released into the extracellular space as a 40 kDa zymogen (Doran et al., 1999). The SpeB zymogen can either be autocatalytically cleaved into the mature enzyme or be cleaved by the mature enzyme itself. Over the maturation process, up to eight intermediate forms of SpeB can be found (Doran et al., 1999; Chen et al., 2003).

Here, we show that in an *ldh* deletion strain of *S. pyogenes* M49 strain 591, the activation of the SpeB zymogen into the mature active SpeB enzyme is hampered. Full virulence and a complete regain of SpeB activity is achieved by extrachromosomal expression of the *ldh* gene in the *ldh* deletion background. The missing SpeB activity likely contributes to the reduced virulence of the M49 *ldh* deletion strain. In accordance with our findings, it has previously been shown that SpeB promotes survival of *S. pyogenes* in human blood (Terao et al., 2008) and that the absence of SpeB renders *S. pyogenes* more prone to phagocytosis by neutrophils (Lukomski et al., 1998). In accordance with that, GAS M49 mutants lacking active SpeB have a decreased virulence in murine intraperitoneal and skin infection models (Lukomski et al., 1997, 1998, 1999; Meinert Niclasen et al., 2011).

Next to the impact on susceptibility toward phagocytosis, another virulence mechanism associated with SpeB is the ability to cleave HK, with the release of pro-inflammatory kinins, independent from PK activation (Herwald et al., 1996). The data of the present study show that upon *ldh* deletion the bacteria have a reduced PK/FXII activity and reduced HK cleavage on their surface. Both—PK and SpeB—efficiently cleave HK, resulting in release of BK, which leads to increased vascular permeability promoting spread of the infection and providing nutrients to growing bacteria (Ben Nasr et al., 1997). Thus reduced PK/FXII activity in combination with the lack of SpeB activity reduces HK cleavage and may further contribute to a reduced virulence.

In addition to the decreased virulence due to the loss of SpeB activity, the missing ability of lactate fermentation might represent a metabolic disadvantage in blood or the larvae. For *Streptococcus pneumoniae* an *ldh* deletion strain has been described to be avirulent in an intravenous mouse infection model (Gaspar et al., 2014). The authors attribute the loss in virulence to a decreased fitness of the *S. pneumoniae ldh* deletion strain, since the bacteria are forced to use the inefficient mixed acid branch of pyruvate metabolism in the absence of lactate dehydrogenase. This is also reflected by a general growth deficiency of the *S. pneumoniae ldh* deletion strain in laboratory media (Gaspar et al., 2014). In *S. pyogenes* the *ldh* deletion also leads to a shift from homofermentative lactate production to mixed acid fermentation (**Figure 1B**) (Fiedler et al., 2011). The *ldh* deletion, however, had only marginal effects on growth efficiency in laboratory media, a finding that also holds for other homofermentative lactic acid bacteria, i.e., *Enterococcus faecalis* and *Lactococcus lactis* (Fiedler et al., 2011). Therefore, a general growth deficiency of the *S. pyogenes ldh* deletion strain cannot be held responsible for the loss in virulence of this strain. Anyway, it still cannot be excluded that the *ldh* deletion represents a disadvantage in terms of metabolic fitness in infection relevant conditions.

The main question remaining is how the *ldh* deletion interferes with SpeB maturation. Hampered SpeB activation has also been reported by Cho and Kang (2013) for a c-di-AMP phosphodiesterase mutant of *S. pyogenes* HSC5, a M14 strain. As in the study of Cho and Kang (2013), the underlying mechanism of interference with SpeB maturation remains obscure and needs further investigation.

## Author Contributions

SO-H, BK, and TF planned the experiments. SM, JW, and LN did the experiments. SO-H, TF, and SM analyzed the data. TF wrote the manuscript. SO-H, SM, and BK edited the manuscript.

## Conflict of Interest Statement

The authors declare that the research was conducted in the absence of any commercial or financial relationships that could be construed as a potential conflict of interest.
