# Supplementary material for: Deletion of the L-Lactate Dehydrogenase Gene ldh in Streptococcus pyogenes Leads to a Loss of SpeB Activity and a Hypovirulent Phenotype
Source: Front Microbiol. 2017 Sep 21;8:1841. doi: 10.3389/fmicb.2017.01841 (PMC5613712; doi:10.3389/fmicb.2017.01841)
Supplement: Supplementary file 1 [file Table_1.PDF]

Supplementary Material

Deletion of the L-lactate dehydrogenase gene *ldh* in *Streptococcus pyogenes* leads to a loss of SpeB activity and a hypovirulent phenotype

Sonja Oehmcke-Hecht[1], Leif Eric Nass[1], Jan Bodo Wichura[1], Stefan Mikkat[2], Bernd Kreikemeyer[1], Tomas Fiedler[1,]*

*** Correspondence:** tomas.fiedler@med.uni-rostock.de

Supplemental table 1: nanoLC-HDMS^E data for proteins mentioned in the manuscript.

					Amount (fmol)						Comparison WT vs Δldh		Comparison WT vs Δldh::ldh		Comparison Δldh vs Δldh::ldh	
					WT		Δldh		Δldh::ldh			Fold change		Fold change		Fold change
**Accession**	**Description**	**Gene name**	**Unique peptides**	**Confidence score**	**Repl 1**	**Repl 2**	**Repl 1**	**Repl 2**	**Repl 1**	**Repl 2**	**Anova (p)**	**WT/Δldh**	**Anova (p)**	**WT/Δldh::ldh**	**Anova (p)**	**Δldh/Δldh::ldh**
B5XLJ8	L-lactate dehydrogenase	ldh	17	165.5	28.4	21.3	1.0	1.0	22.1	20.1	2.31E-03	24.38	4.20E-01	1.18	2.95E-04	0.05
A0A0H3BZJ0	Putative pyruvate formate-lyase	pfl	45	572.7	15.7	15.6	57.5	58.7	14.9	16.5	6.77E-05	0.27	9.91E-01	1.00	1.56E-03	3.71
A0A0H3BVD7	Aldehyde-alcohol dehydrogenase	adhE	50	560.9	5.0	6.3	94.5	93.9	6.2	6.8	1.64E-03	0.06	3.71E-01	0.87	2.62E-04	14.53
B5XJD9	Acetate kinase	ackA	18	153.4	6.5	6.6	8.2	8.2	6.8	7.1	1.89E-04	0.80	8.06E-02	0.94	1.19E-02	1.18
B5XLV6	ATP-dependent 6-phosphofructokinase	pfkA	21	201.5	15.5	14.6	16.7	16.9	16.1	16.0	7.45E-02	0.90	1.66E-01	0.94	3.10E-02	1.04
A0A0H3C1H3	Fructose-bisphosphate aldolase class II	fba	14	187.4	33.8	29.5	23.0	23.5	26.9	25.9	4.80E-02	1.36	1.27E-01	1.20	2.66E-02	0.88
A0A0H3BYT6	Non-phosphorylating glyceraldehyde-3-phosphate dehydrogenase (NADP)	gapN	28	256.8	28.6	20.3	30.9	29.8	25.3	26.4	3.14E-01	0.81	7.24E-01	0.95	3.03E-02	1.17
B5XKM7	Enolase	eno	32	398.3	24.9	13.1	34.5	36.9	26.9	23.8	1.68E-01	0.53	4.07E-01	0.75	3.90E-02	1.41
A0A0H3BYL1	Pyruvate kinase	pyk	40	496.3	20.8	18.3	29.8	28.3	27.8	26.2	2.92E-02	0.67	4.54E-02	0.73	1.92E-01	1.08
A0A0H3C052	NADH oxidase	nox1	19	173.2	16.8	16.3	24.2	24.9	17.1	17.5	2.59E-03	0.67	1.42E-01	0.96	2.60E-03	1.42
A0A0H3C0L8	DNA gyrase subunit A	gyrA	39	379.4	15.1	13.0	12.5	12.5	11.7	11.8	2.65E-01	1.12	1.42E-01	1.19	2.26E-03	1.06
A0A0H3C119	Endopeptidase degP	degP	12	145.8	12.3	12.8	11.0	10.5	11.3	11.5	3.23E-02	1.17	4.05E-02	1.10	1.31E-01	0.94
A0A0H3C2F5	Antiphagocytic M protein	emm49	20	220.6	7.1	6.1	6.3	6.7	12.2	12.5	8.69E-01	1.02	1.63E-02	0.53	2.64E-03	0.52
