# Supplementary material for: Deletion of the L-Lactate Dehydrogenase Gene ldh in Streptococcus pyogenes Leads to a Loss of SpeB Activity and a Hypovirulent Phenotype
Source: Front Microbiol. 2017 Sep 21;8:1841. doi: 10.3389/fmicb.2017.01841 (PMC5613712; doi:10.3389/fmicb.2017.01841)
Supplement: Supplementary file 2 [file Image_1.PDF]

Supplementary Material

Deletion of the L-lactate dehydrogenase gene *ldh* in *Streptococcus pyogenes* leads to a loss of SpeB activity and a hypovirulent phenotype

Sonja Oehmcke-Hecht[1], Leif Eric Nass[1], Jan Bodo Wichura[1], Stefan Mikkat[2], Bernd Kreikemeyer[1], Tomas Fiedler[1,]*

*** Correspondence:** tomas.fiedler@med.uni-rostock.de

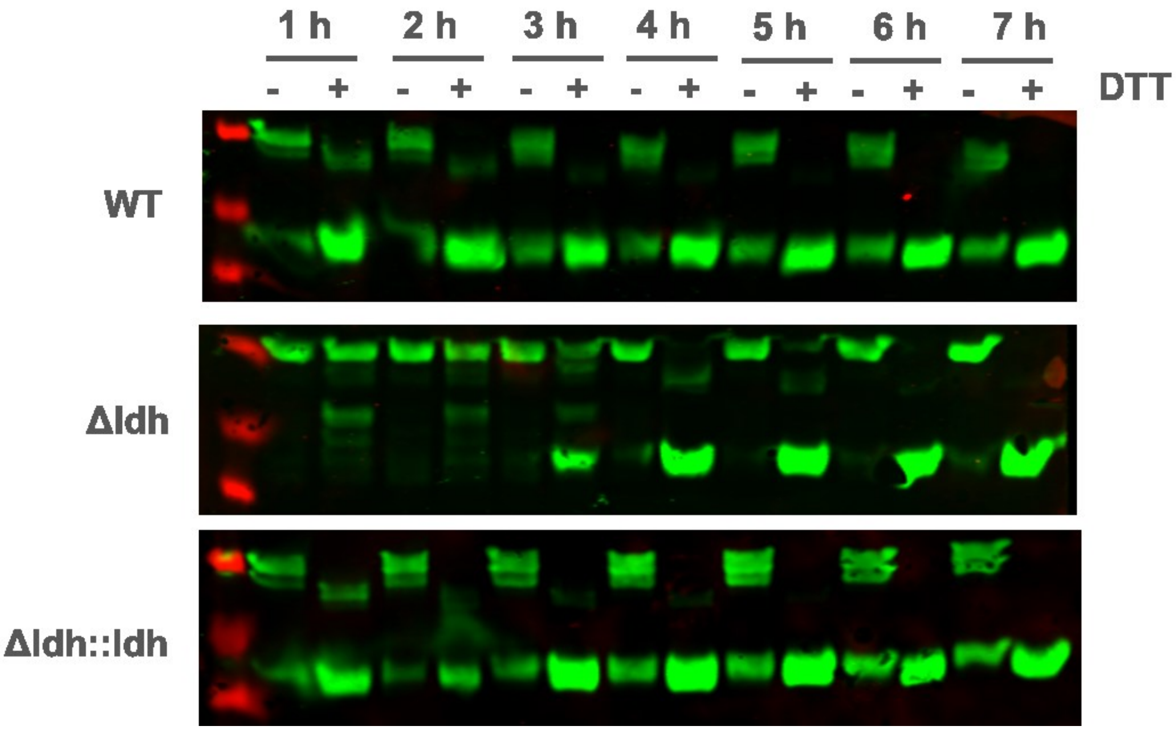

Supplementary Figure 1. Western Blots with SpeB-specific antibodies showing the time-course of SpeB maturation in under reducing (+ DTT) and non-reducing (- DTT) conditions.
