# Supplementary material for: Deletion of the L-Lactate Dehydrogenase Gene ldh in Streptococcus pyogenes Leads to a Loss of SpeB Activity and a Hypovirulent Phenotype
Source: Front Microbiol. 2017 Sep 21;8:1841. doi: 10.3389/fmicb.2017.01841 (PMC5613712; doi:10.3389/fmicb.2017.01841)
Supplement: Supplementary file 3 [file Image_2.PDF]

*Supplementary Material*

# Deletion of the L-lactate dehydrogenase gene *ldh* in *Streptococcus pyogenes* leads to a loss of SpeB activity and a hypovirulent phenotype

Sonja Oehmcke-Hecht[1], Leif Eric Nass[1], Jan Bodo Wichura[1], Stefan Mikkat[2], Bernd Kreikemeyer[1], Tomas Fiedler[1,]*

*** Correspondence:** tomas.fiedler@med.uni-rostock.de

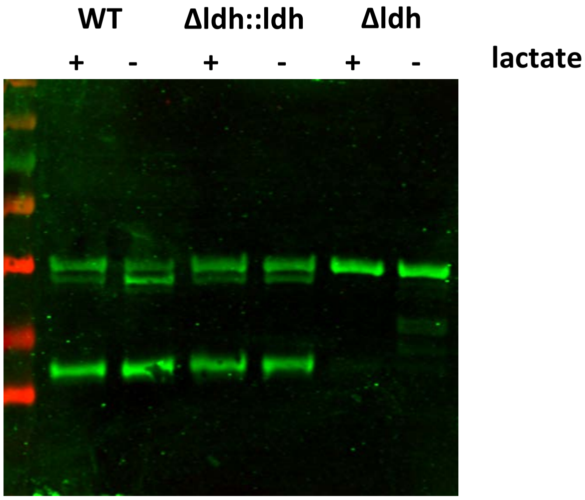


**Supplementary Figure 2.** Western Blot with SpeB-specific antibodies showing the SpeB maturation under reducing conditions upon addition of 30 mM L-lactate to the culture supernatants.
